# A rebrand for proteasome inhibition in solid tumors via continuous hepatic artery infusion

**DOI:** 10.1172/jci.insight.199200

**Published:** 2025-11-04

**Authors:** Carolina M. Larrain, Jack H. Victory, Priyanka P. Desai, Lindsay R. Friedman, Hannah Stepp, Rachel Ashe, Kirsten Remmert, Surajit Sinha, Emily C. Smith, Nicole Russell, Tracey Pu, Alyssa V. Eade, Justine F. Burke, Jason Ho, Michael B. Yaffe, David E. Kleiner, Keith Schmidt, William D. Figg, Jonathan M. Hernandez

**Affiliations:** 1 Surgical Oncology Program; 2 Genitourinary Malignancies Branch, and; 3 Laboratory of Pathology, National Cancer Institute, NIH, Bethesda, Maryland, USA.

**Keywords:** Hepatology, Oncology, Colorectal cancer

## Abstract

Targeting tumors with proteosome inhibitors has demonstrated antitumor activity and has been successfully translated to the clinic for patients with multiple myeloma. However, in patients with solid tumors, treatment with proteosome inhibitors as single agents has consistently failed to yield meaningful responses. In our study, we investigate the potential of hepatic artery infusion pump delivery of carfilzomib, to continuously direct a large dose to the tumor with least hepatic toxicity.

**To the Editor:** Targeting protein homeostasis with proteasome inhibitors has demonstrated antitumor activity across multiple preclinical cancer models, and has been successfully translated to the clinic for patients with multiple myeloma (MM) (1). However, in patients with solid tumors, treatment with proteosome inhibitors as single agents has consistently failed to yield meaningful responses (2). It is well documented that irreversible proteosome inhibitors are susceptible to proteasome recovery via new proteasome synthesis (3).

Continuous drug dosing with arterial infusion pumps is often employed for hepatic malignancies given that tumors derive blood supply (95%) from the hepatic artery (4). Ideal drug candidates for hepatic artery infusion (HAI) pumps have high first-pass metabolism or short plasma half-life, minimizing extrahepatic exposure. Drugs must be soluble (reservoir = 30 mL), stable at body temperature, and compatible with the solvents, anticoagulants, and materials used in the pump. Here, we investigate the potential of HAI pump delivery of carfilzomib (CFZ), an irreversible 20S proteasome inhibitor.

To begin, we determined a mean plasma half-life for intravenously administered CFZ of 16.8 minutes in 45 patients with MM (ClinicalTrials.gov NCT01402284). Further evaluation of plasma CFZ concentrations were used to determine the mean time of last measurable concentration (Tlast) to be 1.85 hours (LC-MS/MS lower limit of quantitation was 0.3 ng/mL) (Figure 1A and Supplemental Table 1; supplemental material available online with this article; https://doi.org/10.1172/jci.insight.199200DS1). The mean total CFZ plasma exposure to the Tlast (calculated using mean AUC_LAST_, mean T_LAST_, and an estimated 3.5 L of plasma for an average human) after dosing was approximately 2% of the average total dose administered intravenously. We attributed the high clearance of CFZ to rapid tissue distribution and deactivation by peptidases/epoxidases in plasma, which limits solid tumor penetration (5).

To assess CFZ compatibility with an HAI pump, we first addressed solubility and stability. The maximum solubility in 5% dextrose in water (D5W) with heparin was determined to be 3 mg/mL. To evaluate stability in contact with titanium, silicone, glass, and polyvinylidene fluoride, we filled the pump reservoir with CFZ (3 mg/mL) and placed the pump in a 37°C incubator to simulate precise contact times. Three sequential aliquots of catheter effluent were collected daily for 14 days and measured using LC-MS/MS. As shown in Figure 1B, the degradation of CFZ was gradual, reaching 68% on day 14.

To measure extrahepatic exposure with HAI, we perfused an intact porcine liver ex vivo on our open-source perfusion system, as previously described (6). Stability of the perfused liver was established prior to the introduction of the drug (Supplemental Figure 1, A–E). CFZ, metformin (low hepatic metabolism control), and floxuridine (high hepatic metabolism control) were then administered consecutively for 120 minutes each. As expected, floxuridine demonstrated an extremely high hepatic extraction of 0.95 (Supplemental Figure 1F), while the hepatic extraction ratio for metformin was 0.62 (Supplemental Figure 1G). Importantly, CFZ demonstrated a hepatic extraction of 0.96 (Figure 1C).

To directly assess the effects of CFZ on colorectal tumors, we first utilized patient-derived organoids (PDOs) obtained from colorectal cancer liver metastases (CRLMs). CRLM PDOs were treated with various doses of CFZ for 1 hour followed by washout to simulate intravenous administration, or for 96 hours to simulate delivery provided by the HAI pump. All samples were assayed after 96 hours of incubation using ATP quantification as a surrogate for cell viability. The IC_50_ for 1 hour of CFZ exposure ranged from 612 nM to 1.22 μM versus 11.9 nM to 34.3 nM for 96 hours of continuous CFZ exposure (Figure 1D and Supplemental Figure 2A). Similar results were obtained using cholangiocarcinoma PDOs (Supplemental Figure 2B).

Next, 2 CRLM PDOs were processed for proteasome activity and cell viability with a 1-hour pulse or 48 hours of continuous CFZ exposure. Following 1 hour of 30 nM CFZ exposure, proteasome activity was readily suppressed (<50% of control), although the activity began to recover significantly by 12–24 hours. No decrease in cell viability was observed in response to a 1-hour pulse treatment (Figure 1, E and F). Similar results were seen with 100 nM CFZ (Supplemental Figure 3, A and B). In contrast, 48 hours of continuous exposure to 30 nM CFZ resulted in sustained suppression of proteosome activity (<10% of control by 6 hours) with significant reduction in cell viability starting 24 hours, with noticeably compromised organoid morphology (Figure 1, G and H, and Supplemental Figure 3, C and D). We noted similar findings with 10 nM CFZ, although 1 nM CFZ showed no significant effects (Supplemental Figure 3, E–H).

Finally, we sought to utilize ex vivo perfusion of human tumor-bearing liver (Figure 1I). Arterial CFZ infusion (3 mg/mL at a rate consistent with HAI pump delivery, 1.3 mL/day) was instituted once stability was established (Supplemental Figure 4, A–D). Consistent with porcine data, CFZ extraction during the run was determined to be 0.98 (Supplemental Figure 4E). Following 48 hours CFZ infusion, we observed a 38% reduction in proteasome activity (Figure 1I), with a concomitant decrease in tumor cell proliferation (Supplemental Figure 4F) and an increase in tumor cell apoptosis without demonstrable effects on the adjacent normal hepatic parenchyma (Figure 1J). The results were confirmed using a second human tumor-bearing liver (Supplemental Figure 4, G and H). These data support a forthcoming phase I trial in patients with previously implanted HAI pumps.

## Funding support

This work is the result of NIH funding, in whole or in part, and is subject to the NIH Public Access Policy. Through acceptance of this federal funding, the NIH has been given a right to make the work publicly available in PubMed Central.

- NIH Intramural Research Program.
- The S. Ritterbush Fund.
- MIT Center for Precision Cancer Medicine.

## Supplementary Material

Supplemental Methods, Figures, and Table 1

Supplemental table 1

Supporting data values

## Figures and Tables

**Figure 1:**
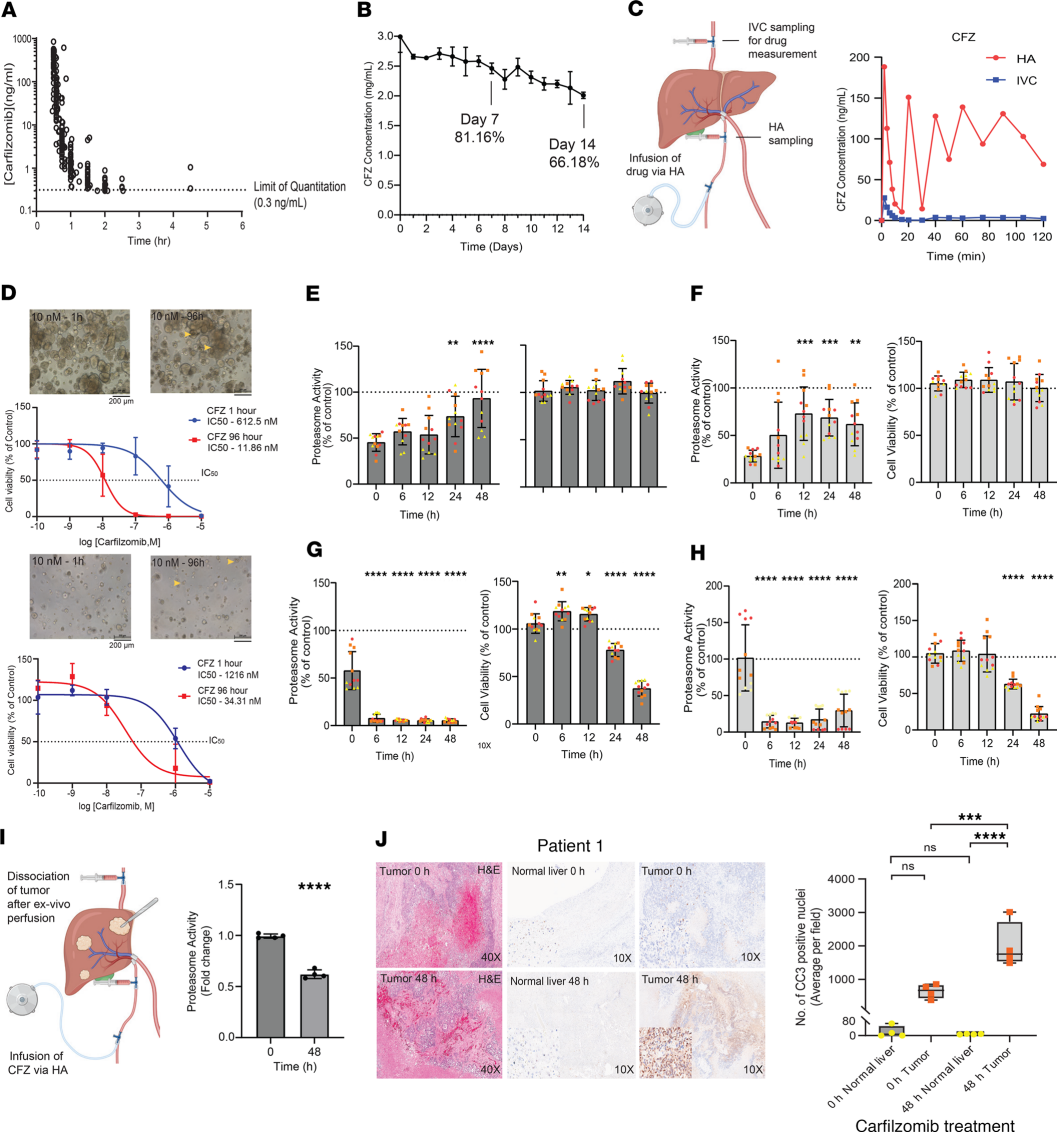
Carfilzomib via HAI. (**A**) CFZ concentrations following 30-minute infusion (*n* = 45). (**B**) CFZ effluent from HAI pump. (**C**) Left: Perfusion schema. Right: Pre- and posthepatic CFZ concentrations. (**D**) Bright-field images and IC_50_ curves for PDO-1 (top) and PDO-2 (bottom) at 10 nM CFZ for 1 hour or 96 hours. Note morphology (yellow arrows). Scale bars: 200 μm. (**E** and **F**) Proteasome activity and cell viability after 1 hour of 30 nM CFZ in PDO-1 (**E**) and PDO-2 (**F**). (**G** and **H**) Proteasome activity and cell viability after 48 hours of 30 nM CFZ in PDO-1 (**G**) and PDO-2 (**H**). Color indicates replicates. (**I**) Left: Perfusion schema. Right: Proteasome activity in tumor at 0 hours and 48 hours of perfusion with CFZ. (**J**) H&E and cleaved caspase-3 (CC3) staining at 0 hours and 48 hours of CFZ treatment. The box-and-whisker plot shows the median (line in box), 25th and 75th percentiles (box bounds), and minimum/maximum (whiskers). *n* = 4. Right: Average of CC3-positive nuclei (original magnification, ×10). Mean ± SD, *n* = 3. Scale bars: 200 μm. *P < 0.05; **P < 0.01; ***P < 0.001; ****P < 0.0001 by 1-way ANOVA followed by Dunnett’s post hoc test (**E**–**H** and **J**) or 2-tailed Student’s *t* test (**I**).

## Version 1 11/04/2025

In-Press Preview

## Version 2 12/22/2025

Electronic publication

## Version 3 01/06/2026

Missing supplemental material was added
