# Supplementary material for: A rebrand for proteasome inhibition in solid tumors via continuous hepatic artery infusion
Source: JCI Insight. 2025 Nov 4;10(24):e199200. doi: 10.1172/jci.insight.199200 (PMC12890499; doi:10.1172/jci.insight.199200)
Supplement: Supplemental Methods, Figures, and Table 1 [file jciinsight-10-199200-s226.pdf]

Methods and Supplemental Figures

Sex as a biological variable:

Sex was not considered a biological variable for the purposes of this study.

Quantification of Carfilzomib in Multiple Myeloma patient plasma

Multiple myeloma patients were administered with 20 mg/m^2 CFZ as a 30 minutes intravenous infusion [NCT01402284]. Blood was collected for pharmacokinetic measurement at different time points including first-dose CFZ (when given alone) at pre-dose, end of 0.5-hour infusion (EOI), (5, 15, 30, 60, 90) minutes, (2, 4, 6, and 24) hours post EOI. Further, plasma concentrations of CFZ were quantified simultaneously using a validated LC-MS/MS assay with a lower limit of quantitation of 0.3 ng/mL.

Liquid Chromatography-Tandem Mass Spectrometry (LC-MS/MS)

Drug concentrations were quantitated via LC-MS/MS using bioanalytical assays specific to the analyte (carfilzomib, floxuridine, metformin) and matrix type (plasma, tissue, bile); each bioanalytical method was validated per the FDA Guidance for Industry for Bioanalytical Method Validation. [$^2H_8$]-carfilzomib, [$^2H_6$]-metformin, and 5-fluorouracil were used as internal standards for quantification for carfilzomib, metformin, and floxuridine, respectively. The calibration ranges were as follows: 0.3 to 300 ng/mL for carfilzomib, 10 to 5000 ng/mL for floxuridine, and 8 to 8000 ng/ml for metformin.

Measurement of drug solubility and stability in the hepatic artery infusion pump

The maximum solubility of CFZ (Kyprolis®, Onyx Pharmaceuticals, Inc., San Francisco, CA, USA) was determined by serially adding sterile dextrose 5% in water (D5W) with heparin (10,000 units/mL, Hospira, Inc, Lake Forrest, IL, USA) at final concentration of 1000 units/mL to lyophilized powder until precipitate was fully dissolved. Starting at a concentration of 5.0mg/mL, diluent was incrementally added until the maximum concentration of CFZ was determined to be 3.0mg/mL. Using this concentration, a hepatic artery infusion pump (INTERA, Austin, TX, USA) was flushed and filled with 30mL (90mg CFZ and 30,000 units of heparin in D5W), prior to placement in a sterile cell culture incubator at 37°C. Three sequential aliquots of catheter effluent were then collected immediately (time 0) followed by every 24 hours over the course of 14 days. Concentration of CFZ was determined via LC-MS/MS. At the completion of 14 days, the catheter reservoir was emptied of remaining solution.

Perfusion of whole porcine liver for measurement of hepatic extraction

First-pass pharmacokinetics were evaluated through the use of whole porcine livers, which underwent *ex vivo* perfusion on our open-source perfusion system. Type-matched porcine blood was used. Once hepatic normothermic perfusion and physiologic parameters were met (defined by oxygen extraction, bile production, as well as physiologic blood flow rates and pressure), drug infusion in the hepatic artery was begun through the use of a programmable continuous syringe pump. Metformin Hydrochloride (Selleck

Chemicals, S1950) dosing was calculated based on a total dose of 1000mg/day, infused at 41.7mg/hour in sterile water. FUDR (Selleck Chemicals, S1299) dosing was calculated based on a total dose of 0.6mg/kg/day, infused at 2mg/hour. CFZ was calculated based on a total dose of 4.2mg/day, infused at 0.175 mg/hour. Drugs were infused for a total of 120 minutes, with a 60 minutes washout period prior to initiation of the subsequent infusion. For sample collection, 1 mL whole blood samples were collected from the proper hepatic artery and inferior vena cava at serial timepoints (0, 2, 4, 6, 8, 10, 15, 20, 30, 40, 50, 60, 75, 90, 105, 120, 135, 150, 165, and 180 minutes). Whole blood samples were centrifuged at 2,000 x g for 90 seconds, plasma supernatant was collected, and samples were immediately transferred to -80℃. Drug concentration was measured using LC-MS/MS. Hepatic extraction ratios were approximated via area under the curve (AUC) calculations derived from drug concentration-time curves for the Hepatic artery (HA) and inferior vena cava (IVC) samples [$(AUC_{HA}-AUC_{IVC})/AUC_{HA}$].

Patient-derived organoids and organoid culture

Patient-derived colorectal adenocarcinoma organoids (919269-233-R1-V3-organoid - PDO-1, 981439-253-R-V1-organoid - PDO-2, 191243-178-R-V1-organoid - PDO-3, and intrahepatic cholangiocarcinoma organoids (819968-124-R3-V1-organoid - PDO-4, 328634-206-R-V1-organoid - PDO-5, 295898-268-R4-V3-organoid – PDO-6) were obtained from the Patient-Derived Models Repository (PDMR) situated at the National Cancer Institute (NCI). 981439-253-R-V1-organoid was cultured in advanced Dulbecco's Modified Eagle Medium (DMEM)/F12 (Invitrogen; 12634-028), supplemented with 10 mM HEPES, 1X GlutaMax supplement (Life Technologies; 35050061), 0.1 mg/mL primocin (InvivoGen; Ant-pm-2), L-WRN conditioned media (1), 1.25 mM N-acetylcysteine (Sigma; A9165-5G), 10 mM nicotinamide (Sigma; N0636-100G), 1X B-27 supplement (R&D Systems; AR008), 1X N-2 supplement (R&D Systems; AR009), and 10 μM Y-27632 dihydrochloride (Tocris; 1254). 919269-233-R1-V3-organoid and 191243-178-R-V1-organoid were cultured in the same culture medium as 981439-253-R-V1-organoid as described above with additional supplementation of 50 ng/mL epidermal growth factor recombinant human protein (hEGF) (Invitrogen; PHG0311-100ug). 819968-124-R3-V1-organoid, 328634-206-R-V1-organoid, and 295898-268-R4-V3-organoid were cultured in the same culture medium as described above, with additional supplementation of 50 ng/mL hEGF, 100 ng/mL fibroblast-growth-factor-10 recombinant human protein (hFGF-10) (R&D Systems: 345-FG), 500 nM SB-431542 (R&D Systems; S1067), and 10 nM gastrin (R&D Systems; 3006). All organoids were negative for human and rodent pathogen testing including mycoplasma contamination. Organoids were suspended in 35 μL reduced growth factor basement membrane extract, type 2 (BME-2) (R&D Systems; 3533-001-02) domes and cultured for 3 days to 2 weeks in 24 well plates in a humidified incubator at 37℃ with 5% CO2 until confluency was reached.

Drug treatment of patient-derived organoids and measurement of cell viability for generation of IC_{50} curves

Organoids were dissociated into single cell suspension and seeded in 96 well plates at approximately 1000 cells per 1 μL BME-2 domes. The cells were cultured for 1-4 days until organoids were formed with duct-

forming morphology which was established under brightfield microscopy. Organoids were exposed to dimethyl sulfoxide (DMSO) as a control or 0.1 nM – 10,000 nM carfilzomib in advanced DMEM/F12 media with supplementation, respective for each organoid as previously described. For the 1 hour carfilzomib treatment group, organoids were exposed to carfilzomib-containing media for 1 hour followed by 2 sequential washes with 1X phosphate-buffered saline (PBS). The media was replaced with the respective advanced DMEM/F12 growth media containing DMSO. For the 96-hour carfilzomib treatment group, organoids were exposed to carfilzomib-containing media for 96 continuous hours. Cell viability following treatment with carfilzomib was determined with CellTiterGlo® reagent per manufacturer's instructions (Promega, Madison, WI, USA; G7572) Luminescence, corresponding to adenosine triphosphate (ATP) from metabolically active cells, was detected with a Cytation 5 multimode microplate reader (BioTek, Winooski, VT, USA). Representative half-maximal inhibitory concentration (IC_{50}) values were calculated using GraphPad Prism version 9.3.1.

Measurement of proteasome activity and cell viability of patient-derived organoids after 1 hour treatment and continuous treatment

Organoids were dissociated into a single cell suspension. 10,000 cells were plated in 2μL BME-2 domes in a 96 well plate. Cells were allowed to grow for 1-4 days until organoids were formed in a duct-forming morphology. For the intermittent (1 hour) treatment group, organoids were treated with either 30nM CFZ or 100nM CFZ in their respective growth media as described above. After 1 hour, the drug containing media was removed and organoids were washed twice with wash media (Advanced DMEM containing HEPES, Glutamine, Fetal Bovine Serum, and antibiotics). Organoids were then incubated in untreated growth media for the duration of the experiment. For the continuous (48 hours) treatment group, organoids were treated with 1nM, 10nM, and 30nM CFZ in their respective growth media for the entirety of the experiment. Controls were treated with the appropriate amount of DMSO for each condition. Organoids were then sequentially processed for measurement of proteasome activity and cell viability at each timepoint, including 0, 6, 12, 24, and 48 hours. Chymotrypsin-like proteasome activity was serially measured using ProteasomeGlo® Cell Based Assay (Promega, Madison, WI, USA; G8661) and cell viability following was determined with CellTiterGlo® reagent as per manufacturer's instructions. Luminescence was measured using Cytation 5 multimode microplate reader (BioTek, Winooski, VT, USA) and representative graphs were plotted using GraphPad Prism version 9.3.1. Each symbol represents n=1(one experiment) and each experiment had 4 replicates. Three different colored symbols represent n=3

Perfusion of partial human livers with CFZ

Tissue was obtained from a patient who underwent clinically indicated resection for histologically confirmed colorectal cancer liver metastases. Segment 3 was resected and underwent *ex vivo* perfusion on our open-source perfusion system using donor patient-matched human blood. The artery, portal inflow, hepatic outflow, and bile duct were cannulated. After physiologic parameters were met, CFZ infusion (3.0mg/mL)

was begun through the hepatic artery with the help of a programmable continuous syringe pump at a rate of 1.3mL/day.

For sample collection, 1 mL whole blood samples were collected from the hepatic artery and hepatic outflow at 0, 2, 4, 6, 8, 10, 15, 20, 30, 40, 50, and 60 minutes. Whole blood samples were then collected hourly for the first 24 hours and every 3 hours for the following 48 hours. The whole blood samples were centrifuged at 2,000 x g for 90 seconds. Plasma supernatant was removed and immediately stored in -80℃. At the end of the experiment, tissue was flushed with normal saline, prior to placement in DMEM media for dissociation or fixation in formalin for FFPE analysis and Hematoxylin and Eosin (H&E) staining.

Measurement of proteasome activity in human tissue after *ex vivo* perfusion with CFZ

After 48 hours of *ex vivo* perfusion and treatment with CFZ, 1 cm^3 of tumor tissue was collected and placed in DMEM for dissociation. For comparison, baseline untreated tissue was collected from the right liver and immediately processed at the time of the initial resection. Tissue was dissociated using the human Tumor Dissociation Kit, following manufacturer's instructions (Miltenyi Biotec, Bergisch Gladbach, Germany; 130-095-929). After dissociation, cells were resuspended and plated in a 96 well opaque white plate, at 10,000 cells per well. Given plates were processed at different time points (baseline tumor was processed immediately after resection vs treatment group was processed 48 hours later after *ex vivo* perfusion), a control (919269-233-R1-V3 CRC organoids) was run on each plate for normalization. 919269-233-R1-V3 organoids were cultured and brought to a single cell suspension as described above and likewise plated at 10,000 cells per well. Chymotrypsin-like proteasome activity was measured using ProteasomeGlo® Cell Based Assay as described above and results were normalized to the control cell line.

Immunohistochemistry staining of cleaved caspase-3 in human tissue after *ex vivo* perfusion with CFZ

Immunohistochemical analysis of Cleaved Caspase-3 (CC3) in a paraffin-embedded liver containing colon tumor was performed using a LeicaBiosystems BondRXm autostainer. The heat-mediated antigen retrieval step was carried out using epitope retrieval (ER) solution 1 (ER-1; Citrate, pH-6.0, LeicaBiosystems #AR9961) for 20 minutes. The primary antibody staining was carried out using anti-rabbit antibody specific for Asp175 residue of CC3 (Dilution 1:400; 9661S; Cell Signaling Technology, Danvers, MA, USA), anti-mouse SQSTM1/p62 (Dilution 1:1000; D5L7G, 88588S; Cell signaling, Danvers, MA, USA), and anti-rabbit Ki67 (Dilution 1:250; D2H10, 9027S; Cell signaling, Danvers, MA, USA) using the Bond Polymer Refine Detection Kit (LeicaBiosystems #DS9800). After removing the slides from the Bond autostainer, slides were dehydrated by using increasing concentrations of ethanol [(70, 95, 100, and 100) %; LeicaBiosystems #3803686)] for 1 minute each followed by xylene (Fischer Scientific #X3RB50) for 1 minute at room temperature, and coverslipped with the xylene based CoverMount solution (Avantik Biogroup #SL6012-A). Further slides were scanned using NanoZoomer S60 (Hamamatsu) and images were viewed using NDP.view.2 software. The quantification of dark brown stained perinuclear CC3 was carried out in four

different fields using Image J (Version 1.54) and statistical analysis was performed using GraphPad Prism (Version 9.3.1).

Statistical analysis

Statistical analyses were performed using GraphPad Prism version 9.3.1. Data are presented as the mean +/- standard deviation (SD). Differences between two groups were compared by Student's *t* test and the relationship between three or more groups was established by One-way ANOVA followed by Dunnett's post hoc test. P-values of less than 0.05 were considered to indicate a significant difference defined by * p≤ 0.05, ** p≤ 0.01, ***p≤ 0.001, ****p ≤ 0.0001; n = 3.

Study Approval:

The study was reviewed and approved by the institutional review board (IRB) for human tissue/livers under the protocol number NCT01915225.This study was conducted in accordance with the guidelines of the Declaration of Helsinki, Belmont report, and U.S. Common Rule.

Data Availability:

The data values of CFZ plasma pharmacokinetic parameters from 45 multiple myeloma patients are reported in the Supporting Data file. Data values shown in graphs and values behind any reported means in the main and supplemental figures are listed in the "Supporting data values" file. Any additional data that support the findings of this study are available from the corresponding author on reasonable request.

Code availability: Access to the system in its entirety is freely available as published in *Friedman et al, 2025, JOVE*, (https://github.com/CCRSOP/OpenSourcePerfusionSystem.git).

Acknowledgements:

This work is the result of NIH funding, in whole or in part, and is subject to the NIH Public Access Policy. Through acceptance of this federal funding, the NIH has been given a right to make the work publicly available in PubMed Central.

Patient derived colorectal adenocarcinoma and intrahepatic cholangiocarcinoma organoids were obtained from the NCI-Patient Derived Models Repository (PDMR) at the National Institutes of Health. This research was supported by the Intramural Research Program of the National Institutes of Health (NIH), the S. Ritterbush Fund, and the MIT Center for Precision Cancer Medicine. The contributions of the NIH author(s) were made as part of their official duties as NIH federal employees, are in compliance with agency policy requirements, and are considered Works of the United States Government. However, the findings and conclusions presented in this paper are those of the author(s) and do not necessarily reflect the views of the NIH or the U.S. Department of Health and Human Services.

Author contribution:

Conceptualization: JMH; Investigation: CML, JHV, PPD, LRF, HS, RA, TP, AE, JFB; Formal analysis: CML, JHV, PPD, LRF, RA, KS, DEK, MBY; Resources: JMH, KS, WDF; Data curation: CML, JHV, PPD, LRF, HS, NR, RA; Project administration: JMH, KS, WDF :Funding acquisition: JMH, WDF ; Writing – original draft: JMH, PPD, MBY, CML, ECS ;Writing – review & editing: JMH, PPD, MBY, KS, KR, WDF, ECS, SS

The sequence of co-first authors was determined based on data contribution.

References:

1. Miyoshi H, and Stappenbeck TS. In vitro expansion and genetic modification of gastrointestinal stem cells in spheroid culture. *Nat Protoc.* 2013;8(12):2471-82.

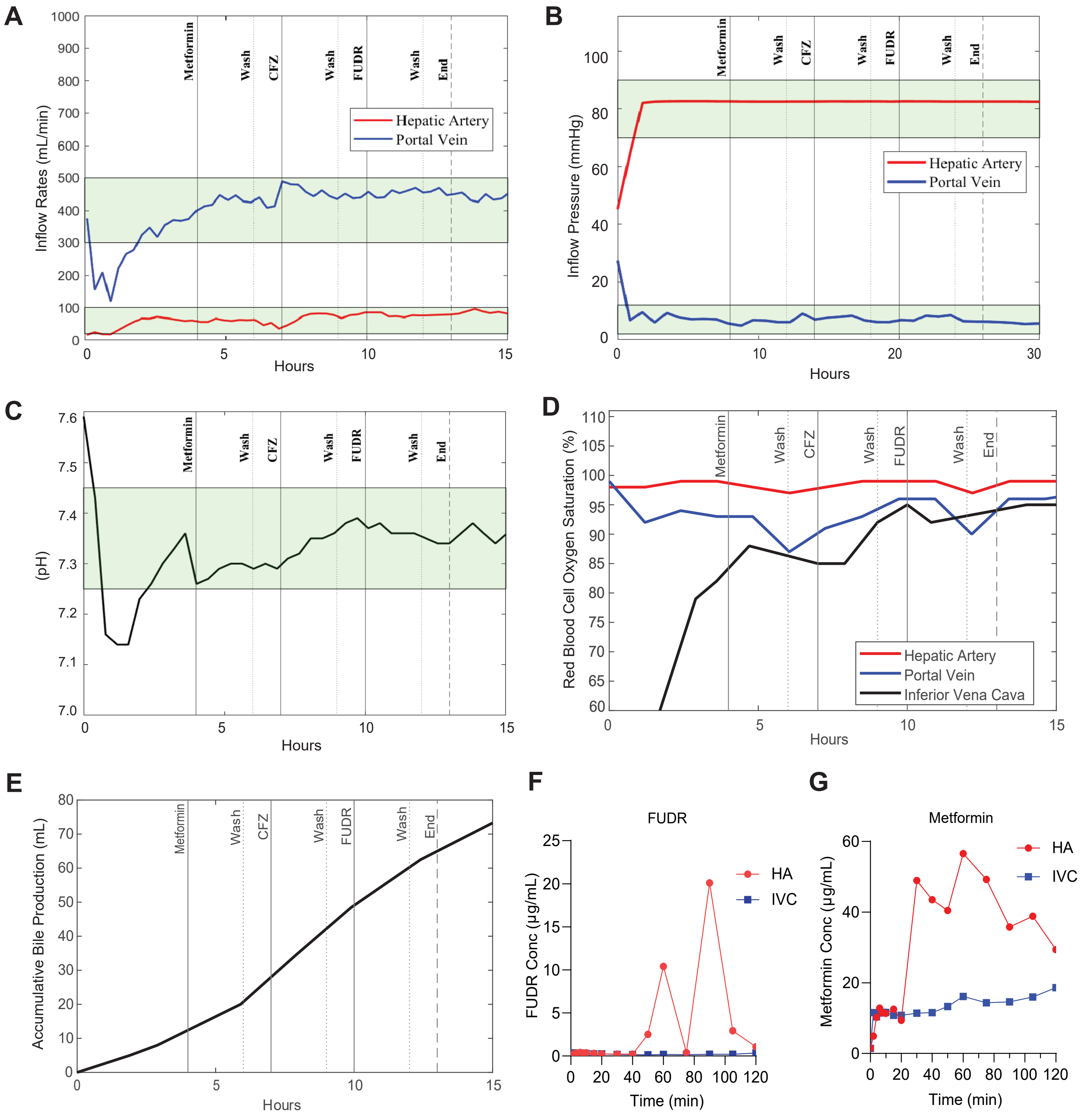
A
Inflow Rates (mL/min)
Metformin
Wash
CFZ
Wash
FUDR
Wash
End
Hepatic Artery
Portal Vein
Hours
B
Inflow Pressure (mmHg)
Metformin
Wash
CFZ
Wash
FUDR
Wash
End
Hepatic Artery
Portal Vein
Hours
C
(pH)
Metformin
Wash
CFZ
Wash
FUDR
Wash
End
Hours
D
Red Blood Cell Oxygen Saturation (%)
Metformin
Wash
CFZ
Wash
FUDR
Wash
End
Hepatic Artery
Portal Vein
Inferior Vena Cava
Hours
E
Accumulative Bile Production (mL)
Metformin
Wash
CFZ
Wash
FUDR
Wash
End
Hours
F
FUDR
FUDR Conc (μg/mL)
HA
IVC
Time (min)
G
Metformin
Metformin Conc (μg/mL)
HA
IVC
Time (min)

Supplemental Figure 1. Establishment of physiologic parameters during *ex vivo* perfusion of a whole porcine liver. (**A**) Plot showing blood inflow rates (mL/min) in the hepatic artery and portal vein during *ex vivo* perfusion of a whole porcine liver prior to drug infusion and during administration of metformin, CFZ, and FUDR for 120 minutes with 60 minutes washout period between administration of each drug. (**B**) Plot of measurement of inflow pressure (mmHg) in the hepatic artery and portal vein. (**C**) Graph showing changes in pH of perfusate overtime as measured by real-time lab values. (**D**) Plot showing red blood cell oxygen saturation in the hepatic artery, portal vein, and inferior vena cava. (**E**) Plot showing total volume (mL) of accumulated bile produced by the liver throughout the experimental run, collected from the canulated common bile duct. Representative graph of *ex vivo* perfusion of whole porcine livers for measurement of pre-hepatic (HA: hepatic artery) and post-hepatic (IVC: inferior vena cava) drug concentration during HA infusion of **(F)** FUDR and **(G)** metformin (120 minutes) for calculation of hepatic extraction (*n* = 1).

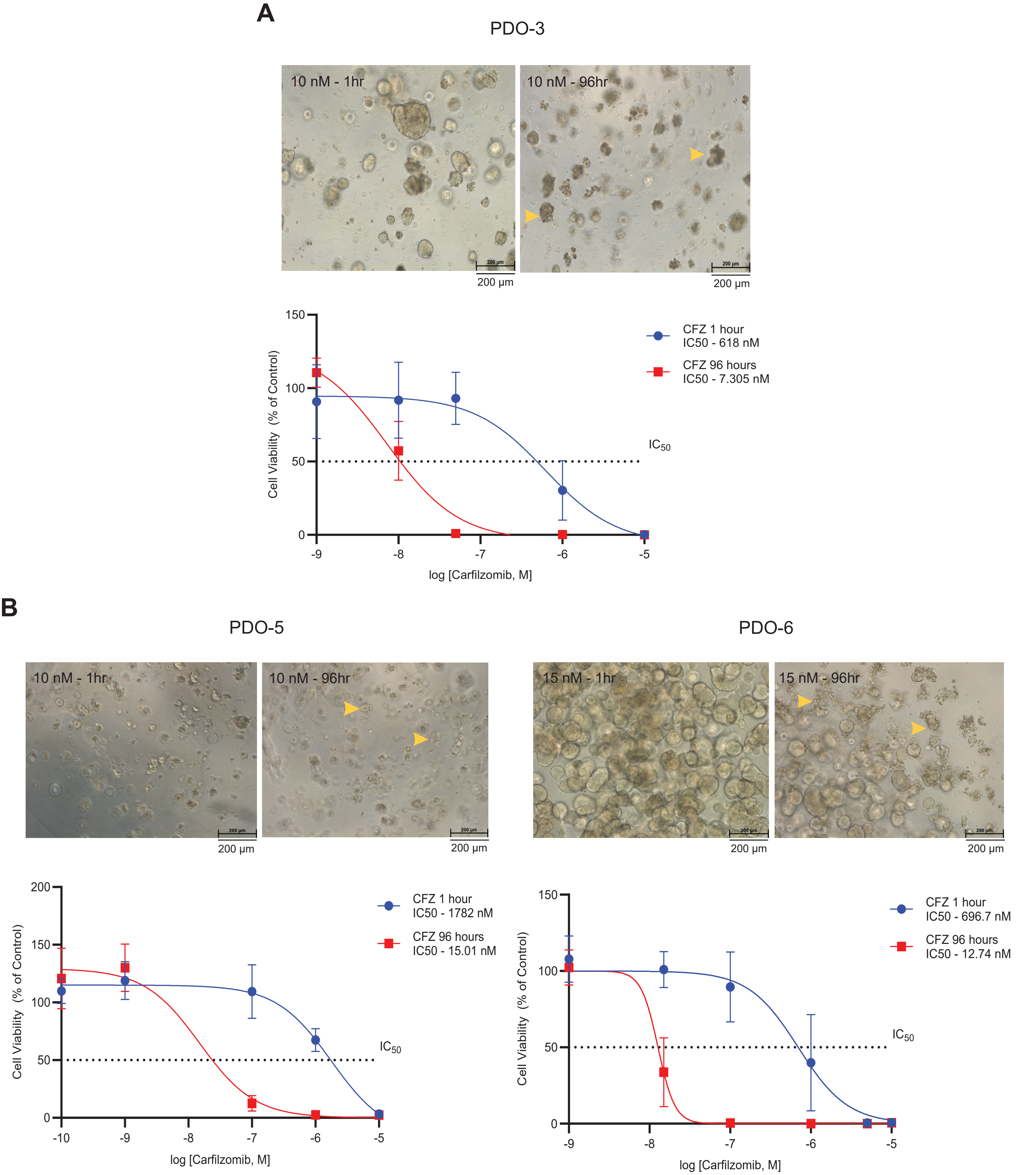
A
PDO-3
10 nM - 1hr
10 nM - 96hr
200 µm
200 µm
Cell Viability (% of Control)
150
100
50
0
-9
-8
-7
-6
-5
log [Carfilzomib, M]
IC50
CFZ 1 hour
IC50 - 618 nM
CFZ 96 hours
IC50 - 7.305 nM
B
PDO-5
10 nM - 1hr
10 nM - 96hr
200 µm
200 µm
Cell Viability (% of Control)
200
150
100
50
0
-10
-9
-8
-7
-6
-5
log [Carfilzomib, M]
IC50
CFZ 1 hour
IC50 - 1782 nM
CFZ 96 hours
IC50 - 15.01 nM
PDO-6
15 nM - 1hr
15 nM - 96hr
200 µm
200 µm
Cell Viability (% of Control)
150
100
50
0
-9
-8
-7
-6
-5
log [Carfilzomib, M]
IC50
CFZ 1 hour
IC50 - 696.7 nM
CFZ 96 hours
IC50 - 12.74 nM

Supplemental Figure 2. IC_{50} values in CRLM and CCA PDOs after pulse and continuous exposure to CFZ. Representative brightfield images and dose response curves with IC_{50} values in (**A**) 1 CRLM PDO line (PDO-3) and (**B**) 2 ICC PDO lines (PDO-5 and PDO-6) after pulse exposure (1h treatment) or continuous exposure (96h treatment) to CFZ (*n* = 3). Yellow arrows pointing towards the compromised organoid morphology. Data represented as mean$\pm$SD. Scale bar = 200 μM.

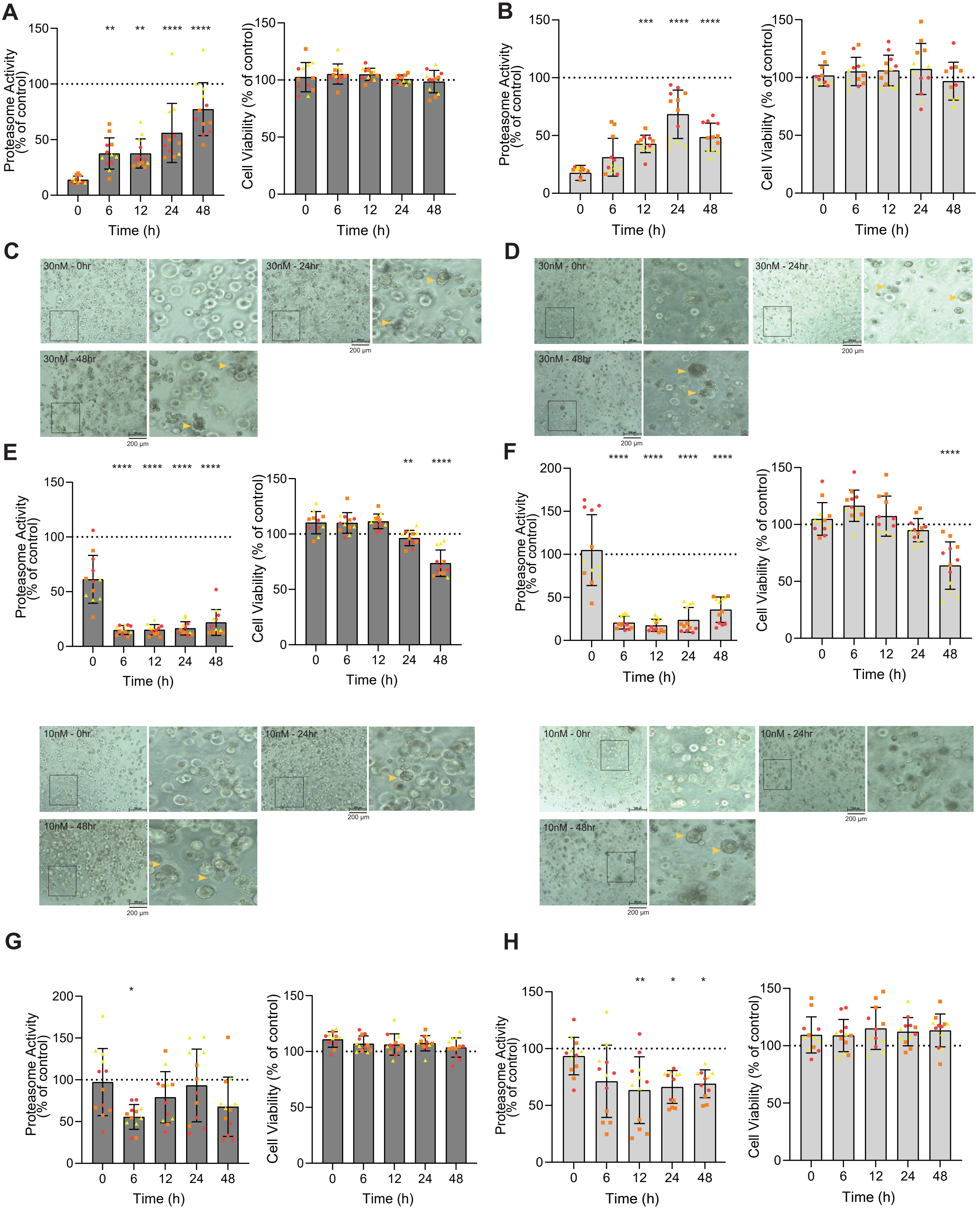
A
Proteasome Activity (% of control)
**
**
****
****
Cell Viability (% of control)
Time (h)
B
***
****
****
C
30nM - 0hr
30nM - 24hr
30nM - 48hr
200 µm
D
30nM - 0hr
30nM - 24hr
30nM - 48hr
200 µm
E
****
****
****
****
**
****
F
****
****
****
****
****
10nM - 0hr
10nM - 24hr
10nM - 48hr
200 µm
G
*
H
**
*
*
0
6
12
24
48
0
50
100
150
200

Supplemental Figure 3. Comparison of proteasome activity and cell viability after intermittent or continuous CFZ exposure. (**A**-**B**) Dot plot showing proteasome activity and cell viability after 1 hour treatment with CFZ at 100nM in PDO-1(**A**) and PDO-2 (**B**) CRLM PDOs. (**C**-**D**) Representative brightfield images after continuous treatment with CFZ at 30nM in PDO-1 (**C**) and PDO-2 (**D**) CRLM PDOs. Yellow arrows indicate compromised organoid morphology. (**E**-**F**) Dot plot showing proteasome activity and cell viability after continuous treatment with CFZ at 10 nM in PDO-1 (**E**) and PDO-2 (**F**) CRLM PDOs with their representative brightfield images. Yellow arrows indicate compromised organoid morphology. (**G**-**H**) Dot plot showing proteasome activity and cell viability after continuous treatment with CFZ at 1 nM in PDO-1 (**G**) and PDO-2 (**H**) CRLM PDOs. Data displayed as mean$\pm$SD. Each color represents one experiment with 4 replicates. Statistical significance measured by One-way ANOVA followed by Dunnett's post hoc test and indicated by * $p \leq 0.05$, ** $p \leq 0.01$, ***$p \leq 0.001$, ****$p \leq 0.0001$; n = 3. Scale bar = 200 μM.

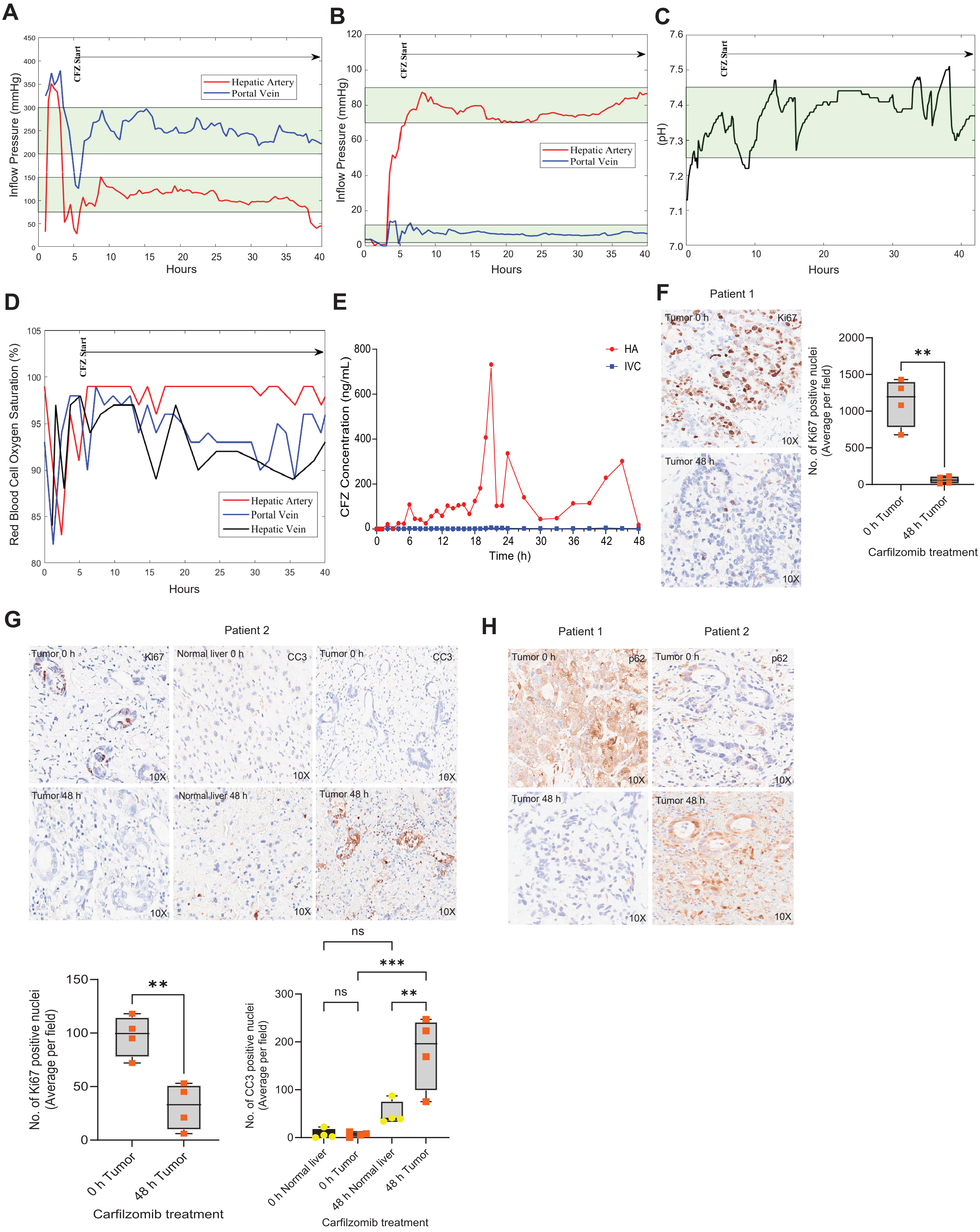

A
CFZ Start
Hepatic Artery
Portal Vein
Inflow Pressure (mmHg)
Hours
B
CFZ Start
Hepatic Artery
Portal Vein
Inflow Pressure (mmHg)
Hours
C
CFZ Start
(pH)
Hours
D
CFZ Start
Red Blood Cell Oxygen Saturation (%)
Hepatic Artery
Portal Vein
Hepatic Vein
Hours
E
HA
IVC
CFZ Concentration (ng/mL)
Time (h)
F
Patient 1
Tumor 0 h
Ki67
10X
Tumor 48 h
10X
No. of Ki67 positive nuclei (Average per field)
**
0 h Tumor
48 h Tumor
Carfilzomib treatment
G
Patient 2
Tumor 0 h
Ki67
Normal liver 0 h
CC3
Tumor 0 h
CC3
Tumor 48 h
Normal liver 48 h
Tumor 48 h
10X
H
Patient 1
Patient 2
Tumor 0 h
p62
Tumor 0 h
p62
Tumor 48 h
Tumor 48 h
10X
No. of Ki67 positive nuclei (Average per field)
**
0 h Tumor
48 h Tumor
Carfilzomib treatment
No. of CC3 positive nuclei (Average per field)
ns
***
ns
**
0 h Normal liver
0 h Tumor
48 h Normal liver
48 h Tumor
Carfilzomib treatment

Supplemental Figure 4. Establishment of physiologic metrics during *ex vivo* perfusion of a human liver segment prior to infusion with CFZ. (**A**) Plot showing blood inflow rates (mL/min) in the hepatic artery and portal vein during *ex vivo* perfusion of segment 3 of a human liver with CRLM. (**B**) Plot of measurement of inflow pressure (mmHg) in the hepatic artery and portal vein. (**C**) Plot showing changes in pH of perfusate overtime. (**D**) Plot showing red blood cell oxygen saturation in the hepatic artery, portal vein, and hepatic vein. **(E)** Representative graph of *ex vivo* perfusion of human livers for measurement of pre-hepatic (HA: hepatic artery) and post-hepatic (IVC: inferior vena cava) drug concentration during HA infusion of CFZ (48 hours) for calculation of hepatic extraction. (**F**) Representative images of Ki67 staining at 0h and after 48h CFZ treatment in hepatic metastasis in patient 1 (Magnification-10X). Right: Graph showing average of Ki67 positive nuclei per four fields (**G**) Representative images of Ki67 and Cleaved caspase-3 (CC3) staining at 0h and after 48h CFZ treatment in normal and hepatic metastasis in patient 2 (Magnification-10X). Right: Graph showing average of Ki67 and CC3 positive nuclei per four fields. (**H**) Representative images of SQSTM1/p62 staining at 0h and after 48h CFZ treatment in hepatic metastasis in patients 1 and 2 (Magnification-10X). Data shown as mean$\pm$SD; * $p \leq 0.05$, ** $p \leq 0.01$, ***$p \leq 0.001$, ****$p \leq 0.0001$; $n = 2$.

Supplemental Table 1: Relevant carfilzomib plasma pharmacokinetic parameters reported from 45 patients with newly diagnosed multiple myeloma.

Dose (ng)	$T_{½}$ (hr)	C_{max} (Ug/L)	T_{max} (hr)	Clast (hr)	Tlast (hr)	AUClast (hr*ug/L)	CL (L/hr)	Vss (L)
38100±4400	0.28±0.17	248±196	0.52±0.05	0.58±0.63	1.85±0.87	112±141	914±1152	358±542^

Number displayed as Mean ± Standard Deviation (SD)

Abbreviations:

$T_{1/2}$	Half-life
C_{max}	Maximum plasma concentration
T_{max}	Time of C_{max}
Clast	Last measurable plasma concentration
Tlast	Mean time of last measurable concentration
AUCLast	Area under the plasma concentration vs time curve up to the last observed time point
CL	Clearance
Vss	Volume of distribution at steady state
^	One data point excluded due to inaccurate calculation
